# Glutathione Transferases Are Involved in the Genotype-Specific Salt-Stress Response of Tomato Plants

**DOI:** 10.3390/antiox12091682

**Published:** 2023-08-28

**Authors:** Edit Horváth, Kitti Kulman, Bernát Tompa, Ádám Barnabás Hajnal, Alina Pelsőczi, Krisztina Bela, Ágnes Gallé, Jolán Csiszár

**Affiliations:** 1 Department of Plant Biology, Faculty of Sciences and Informatics, University of Szeged, H-6726 Szeged, Hungary; kittikulman30@gmail.com (K.K.); tompa_bernat@yahoo.com (B.T.); hadam996@gmail.com (Á.B.H.); pelsoczi.97@gmail.com (A.P.); bela.krisztina@szte.hu (K.B.); gallea@bio.u-szeged.hu (Á.G.); csiszar@bio.u-szeged.hu (J.C.); 2 Agricultural Institute, Centre for Agricultural Research, Eötvös Lóránd Research Network, H-2462 Martonvásár, Hungary; 3 Doctoral School in Biology, Faculty of Science and Informatics, University of Szeged, H-6726 Szeged, Hungary

**Keywords:** glutathione transferases, redox homeostasis, salt stress, *Solanum lycopersicum* L., transcription regulation

## Abstract

Glutathione transferases (GSTs) are one of the most versatile multigenic enzyme superfamilies. In our experiments, the involvement of the genotype-specific induction of *GST* genes and glutathione- or redox-related genes in pathways regulating salt-stress tolerance was examined in tomato cultivars (*Solanum lycopersicum* Moneymaker, Mobil, and Elán F1). The growth of the Mobil plants was adversely affected during salt stress (100 mM of NaCl), which might be the result of lowered glutathione and ascorbate levels, a more positive glutathione redox potential (*E_GSH_*), and reduced glutathione reductase (GR) and GST activities. In contrast, the Moneymaker and Elán F1 cultivars were able to restore their growth and exhibited higher GR and inducible GST activities, as well as elevated, non-enzymatic antioxidant levels, indicating their enhanced salt tolerance. Furthermore, the expression patterns of *GR*, selected *GST*, and transcription factor genes differed significantly among the three cultivars, highlighting the distinct regulatory mechanisms of the tomato genotypes during salt stress. The correlations between *E_GSH_* and gene expression data revealed several robust, cultivar-specific associations, underscoring the complexity of the stress response mechanism in tomatoes. Our results support the cultivar-specific roles of distinct *GST* genes during the salt-stress response, which, along with *WRKY3*, *WRKY72*, *DREB1*, and *DREB2*, are important players in shaping the redox status and the development of a more efficient stress tolerance in tomatoes.

## 1. Introduction

Glutathione transferases (GSTs) are one of the most versatile enzymes, which have a broad range of substrates and participate in various chemical reactions, as well as physiological or developmental pathways [1,2,3]. In plants, they were initially documented as the members of the detoxification routes involved in maize defense against herbicides [4]. GSTs catalyze a wide range of reactions, of which the most well-known is the conjugation of glutathione (γ-Glu-Cys-Gly, GSH) to electrophilic compounds. Several GSTs also display glutathione peroxidase (GPOX) activity; thus, they are involved in the reduction of organic hydroperoxides. However, GSTs can participate in a variety of other reactions, interacting with numerous alternative substrates [2,3,5,6,7]. Several studies have shown that they are involved in the response to various stresses [8,9,10,11,12,13], including salt stress [14,15,16,17,18,19,20,21].

Soil salinization is an increasing threat to agriculture, hampering growth and development of plants and reducing crop yield [22]. The rate of shoot growth decreases immediately if the salt concentration reaches the threshold level. This is mostly the result of the osmotic effect of the salt around the roots [23]. Ion toxicity occurs when the slow accumulation of sodium in the shoot inhibits the metabolic processes, which may lead to a reduction in growth [22]. The inhibited, early-stage plant development decreases the yield and puts at risk the quality and quantity of the final products [24].

Over the last few decades, plant responses to salinity have been studied extensively, and it has been demonstrated that, when the salt concentration reaches a critical level, it causes osmotic stress, which is accompanied by ionic toxicity if the plants cannot maintain ion homeostasis. Primary stresses can induce other secondary stresses, especially oxidative stress [23,25,26].

Different biotic and abiotic stress conditions can lead to an increase in several reactive oxygen species (ROS), such as superoxide (O_2_^−^) or hydrogen peroxide (H_2_O_2_) [27]. These highly reactive molecules may be harmful for the cells, a phenomenon known as oxidative distress; however, plants can utilize ROS as signaling molecules, especially under mild or moderate stress conditions (eustress) [28].

Various essential antioxidant mechanisms have evolved to regulate ROS levels (especially H_2_O_2_), such as the ascorbate–glutathione (AsA–GSH) pathway and its related enzymes [29]. The total amounts of non-enzymatic antioxidants (e.g., GSH, AsA, and flavonoids) and their redox status (ascorbate/dehydroascorbate: AsA/DHA; and reduced/oxidized forms of glutathione: GSH/GSSG) play crucial roles in cellular processes [30,31,32,33]. The preservation of a high GSH level and GSH/GSSG ratio and, thus, a highly negative glutathione redox potential (*E_GSH_*), is important for the efficient functioning of cells and organs [34,35,36]. The maintenance of redox homeostasis and the highly negative *E_GSH_* is achieved through the continuous reduction of GSSG by glutathione reductase (GR) enzymes and/or the increased de novo synthesis of GSH [37,38].

Several reactions may contribute to GSH oxidation during oxidative stress, leading to modifications in the status of this potentially important cellular redox signal. Of the molecules able to oxidize GSH to produce GSSG, the most prominent molecules are the ROS. An outstanding GSSG-generating process is the reduction of DHA to AsA, which is catalyzed by the dehydroascorbate reductase (DHAR) enzyme [39]. However, several studies suggest that other enzymes, like glutaredoxins (GRXs) and GSTs, may also contribute to GSH oxidation to GSSG [39]. Previously, using *Arabidopsis* T-DNA insertional *gst* mutants, we found that several specific AtGSTs participate in the control of the *E_GSH_* by elevating the GSH content of cells [20,40]. Moreover, a cultivar-specific correlation was found between the redox status of tomato roots and the expression of certain *GST* genes in different stress conditions [21].

Based on their protein and gene sequences, plant GSTs can be grouped into 10 classes. Among them, the DHAR, tau (GSTU), and lambda (GSTL) classes are plant-specific [2]. The phi class (GSTF) was also considered to be plant-specific; however, homologue sequences have been discovered in some bacteria and fungi [41]. Generally, the GSTU and GSTF isoenzymes, which show GST and GPOX activities, are the most abundant [3,42,43]. In tomato (*Solanum lycopersicum*) plants, 90 *SlGST* genes were identified and, based on the protein sequences, 57 GSTU, 7 GSTL, 6 GSTF, 6 DHAR, 4 GSTT, 3 EF1Bγ, 2 zeta (GSTZ), 2 glutathionyl-hydroquinone reductase (GHR), 2 microsomal, and 1 tetrachlorohydroquinone dehalogenase (TCHQD) isoenzymes could be found [11]. Earlier studies have identified tau class GSTs (Bax-inhibitor GST or BI-GST and LeGSTU1-5) in tomato plants that were able to mitigate oxidative-stress-induced cell death in yeast cells [44,45]. Ding et al. described the importance of one of them (SlGSTU24) in tomatoes, which showed elevated GST activity in the attenuation of low-temperature-induced oxidative stress [12]. *Arabidopsis* plants overexpressing the *LeGSTU2* tomato gene (current name: *SlGSTU18*) exhibited higher salt- and osmotic-stress tolerance, due to elevated proline content and increased antioxidant capacity [17]. Sun et al. assessed the transcriptomic profiles of Moneymaker plants and a salt-tolerant wild tomato (*Solanum pimpinellifolium* ‘PI365967’) and found that several *GST* genes were expressed at a higher level in the salt-tolerant genotype; however, numerous *SlGST* genes upregulated by salt stress were identified only in Moneymaker plants [46]. A specific set of *GST* genes is induced during a response to salt or osmotic stresses, and elevated GST and/or GPOX activities form part of successful osmotic-, dehydration-, or salt-stress responses [11,15,21]. Furthermore, increased GST and GPOX activities, an accumulation of GSH, and an elevated GSH/GSSG ratio were reported to characterize resilience to stress in tomato plants [21,47].

The aim of this study was to compare the salt-stress responses of three tomato (*S. lycopersicum*) genotypes commonly cultivated in Hungary (cv. Mobil and cv. Elán F1) and extensively used in research (cv. Moneymaker). Experiments were performed to analyze genotype-specific, stress-inducible *SlGSTs*, and glutathione- or redox-related genes, which are involved in the regulation of stress tolerance. Our results indicate that, in these investigated cultivars, various routes can be observed in the achievement of salt-stress tolerance, in which the regulation of the redox status has particular importance.

## 2. Materials and Methods

### 2.1. Plant Material and Growth Conditions

Three tomato cultivars (*Solanum lycopersicum* L. Moneymaker, Mobil, and Elán F1) were used in this study. The seeds were germinated for three days in the dark and were transferred in a perlite-packed tube into plastic containers filled with modified Hoagland solution (Supplementary Figure S1) [15]. The plants were grown in a controlled environment with a light intensity of 200 μmol m^−2^ s^−1^, a light/dark period of 12 h each, day and night temperatures of 24/22 °C, and a relative humidity of 55–60% for four weeks. The plants were treated for one week by adding 100 mM of NaCl to the nutrient solution. The NaCl concentration was selected based on our earlier results [15]. The shoot and root lengths and fresh weights were determined using a ruler and digital scale, respectively. The samples for physiological, biochemical, or molecular biological analysis were collected after one week of treatment from the second, fully expanded, young leaves and roots.

### 2.2. H_2_O_2_ and Malondialdehyde Content Measurements

The H_2_O_2_ content was determined using xylenol orange (XO) reagent, according to Mátai and Hideg [48], with some modifications, as described in Gallé et al. [20]. In short, 100 mg of leaf or root tissue was homogenized on ice with 250 μL of 5% trichloroacetic acid, then centrifuged at 10,000× *g* for 15 min at 4 °C. A 100 μL clear supernatant was added to 1 mL of the XO chromophore solution, which consisted of 0.125 mM of XO, 100 mM of sorbitol, 0.25 mM of (NH_4_)_2_Fe(SO_4_)_2_*6H_2_O, and 25 mM of sulfuric acid. The ferric–XO assay is based on the fact that H_2_O_2_ oxidizes Fe^2+^ to Fe^3+^ at an acidic pH, which forms a colored product with XO, and the reaction is enhanced with sorbitol. The absorbance was measured using a Uvikon 930 spectrophotometer (Uvikon 930 spectrophotometer, Kontron AG, Eching, Germany) at a 570 nm wavelength after 30 min incubation in the dark at room temperature. The quantity of H_2_O_2_ was determined by utilizing a standard curve, prepared with H_2_O_2_ concentrations ranging from 0.01 to 0.1 mM.

Malondialdehyde (MDA) formation was detected using the modified method of Ederli et al. [49], as was published in Gallé et al. [21]. A quantity of 100 mg of leaf or root tissue was homogenized in 1 mL of 0.1% TCA, in the presence of 100 μL of 4% butylhydroxytoluene to prevent further lipid peroxidation, then centrifuged at 10,000× *g* for 20 min at 4 °C. A 250 μL supernatant was assayed by adding 1 mL of 20% TCA containing 0.5% thiobarbituric acid. After incubation at 96 °C for 30 min, the absorbance was measured at 532 nm and corrected for nonspecific absorbance at 600 nm. The concentration of MDA was calculated using an extinction coefficient of 155 mM^−1^ cm^−1^.

### 2.3. Ascorbic Acid and Glutathione Content Determination

Non-enzymatic antioxidant contents (ascorbic acid and glutathione) were determined as described in Bela et al. [50]. A total of 300 mg of leaf or root tissue was homogenized in 1.2 mL of 5% TCA. After centrifugation (10,000× *g*, 20 min, 4 °C), the supernatant was used for further measurements. In the used colorimetric assay, Fe^3+^ was reduced by AsA to Fe^2+^ in an acidic solution, then, Fe^2+^ formed a complex with α-α′-bipyridyl [51]. Since only reduced AsA can be detected by this method, to determine the total ascorbic acid content, 100 μL of supernatant was pre-treated with 10 mM of dithiothreitol (Sigma-Aldrich, Darmstadt, Germany). The ascorbic acid concentration was determined spectrophotometrically at 525 nm. Dehydroascorbate (DHA) content was calculated as the difference between the concentration of total and reduced ascorbate.

The total and oxidized glutathione contents were determined using GR enzymatic assay, measured at 412 nm [21]. Reduced glutathione was masked by pre-treating the samples with 4-vinylpyridine. The content of reduced GSH was calculated by subtracting the concentration of GSSG from the total glutathione concentration. The glutathione (GSH/GSSG couple) reduction potential (*E_GSH_*) was determined with the Nernst equation, according to Schafer and Buettner [52].

### 2.4. Enzyme Activity Determination

The protein contents [53] and enzymatic activities [15,38] were measured spectrophotometrically. A phosphate buffer (0.1 mM, pH 7.0) containing 1% phenylmethylsulfonyl fluoride was used to homogenize 200 mg of fresh leaf or root tissue in the presence of 1 mM of polyvinyl-polypirrolidone on ice, which was then centrifuged at 10,000× *g* for 20 min at 4 °C. The supernatant was used for the enzyme activity assays. The activity of GR (EC 1.8.1.7) was assessed by monitoring the increase in absorbance at 412 nm, as 5,5′-dithio-bis(2-nitrobenzoic acid) (DTNB; Sigma-Aldrich, Darmstadt, Germany) was reduced by GSH, which was produced from GSSG [54]. The specific activity was determined as the amount of reduced DTNB in µmol min^−1^ protein mg^−1^ (ε_420_ = 13.6 mmol L^−1^ cm ^−1^).

The artificial substrate, 1-chloro-2,4-dinitrobenzene (CDNB; Sigma-Aldrich, Darmstadt, Germany), was used to determine the GST (EC 2.5.1.18) activity [55]. A total 1 U was the amount of the enzyme producing 1 nmol of conjugated product in 1 min (ε_340_ = 9.6 mmol L^−1^ cm^−1^).

The GPOX (EC 1.11.1.9) activity was measured with cumene hydroperoxide (CHP; Sigma-Aldrich, Darmstadt, Germany) as a substrate [56]. The decrease in NADPH was followed by measuring the absorbance at 340 nm. The nonspecific NADPH decrease was corrected by using additional measurements without CHP. A total of 1 U was equal to the nmol of converted NADPH in 1 min (ε_340_ = 6.22 mmol L^−1^ cm^−1^).

The DHAR (EC 1.8.5.1) activity was assayed as was published in [15], using the method of Edwards and Dixon [5]. The increase in AsA was followed by measuring the absorbance at 265 nm. The enzyme activity was calculated as the increase in AsA content in nmol min^−1^ mg protein^−1^ (ε_265_ = 14.0 mmol L^−1^ cm^−1^).

### 2.5. RNA Purification and Gene Expression Analyses

High-throughput quantitative real-time PCR (HT-qPCR, Avidin Ltd., Szeged, Hungary) and quantitative real-time PCR (qPCR, qTOWER Real-Time qPCR System, Analytik Jena, Jena, Germany) were applied to detect the expression of *S. lycopersicum* genes in the leaves and roots of the three investigated cultivars after one week of 100 mM NaCl treatment. The genes investigated in this study were selected based on our previous results [15,21] and on the number of W-box elements (TTGAC) found in the 5′ cis regulatory regions of the *GST* genes (Table S1). RNA was extracted from 50–100 mg of tissue using a Quick-RNA Miniprep Kit (Zymo Research, Freiburg, Germany), according to the manufacturer’s instructions. To eliminate genomic DNA, DNase treatment was applied, and a further purification step was added to the procedure (RNA Clean & Concentrator-25 Kit, Zymo Research, Freiburg, Germany). Reverse transcription was executed by using 1 μg of RNA and random hexamers with RevertAid reverse transcriptase. The primers used for the qPCR are given in Table S2. The tomato *actin2* and *elongation factor* genes were used as internal controls for data normalization. The data from the HT-qPCR and qPCR were calculated using the 2^−ΔΔCt^ formula [57]. To demonstrate the variations in gene expression levels due to salt stress, the relative transcript levels were normalized to the control samples for each gene, and then the 2^−ΔΔCt^ values were log2 transformed. The results were then visualized on a heat map. In the untreated plants, the expression levels were normalized to that of the Mobil cultivar for each gene.

### 2.6. Promoter Analysis

In silico screening was performed via NewPLACE [58] in the cis regulator sequences of the 5′ regulatory regions (1500 bp upstream from the start codon) in the selected *GST* genes, sourced from the Sol Genomics Network (SGN) database (http://solgenomics.net, accessed on 1 May 2022). Furthermore, a systematic search in published research papers for the transcription factors (TFs) involved in abiotic-stress responses and salt- or drought-tolerance of tomato was performed. According to the promoter analysis and data found in the literature, eight salt- and osmotic-stress-responsive TFs were selected for further gene expression studies (Supplementary Table S3).

### 2.7. Determination of Pearson’s Correlation Coefficients

Pearson’s correlation coefficients were computed using Microsoft Excel 2010 to assess the association between the direct values of redox potential (*E_GSH_*) and the expression of specific genes (ΔCt values; Supplementary Table S4) [57].

### 2.8. Statistical Analyses

All experiments were repeated at least twice, and the samples originated from three biological replicates. The data presented in this study are the means ± SD from at least three measurements, unless stated otherwise. The statistical analysis was performed using Microsoft Excel 2010 and Student’s *t*-tests (* *p* ≤ 0.05, ** *p* ≤ 0.01, *** *p* ≤ 0.001).

## 3. Results

### 3.1. Compensation of Growth Inhibition and Accumulation of H_2_O_2_ Due to Salinity Are Different between the Tomato Cultivars

**Figure 1:**
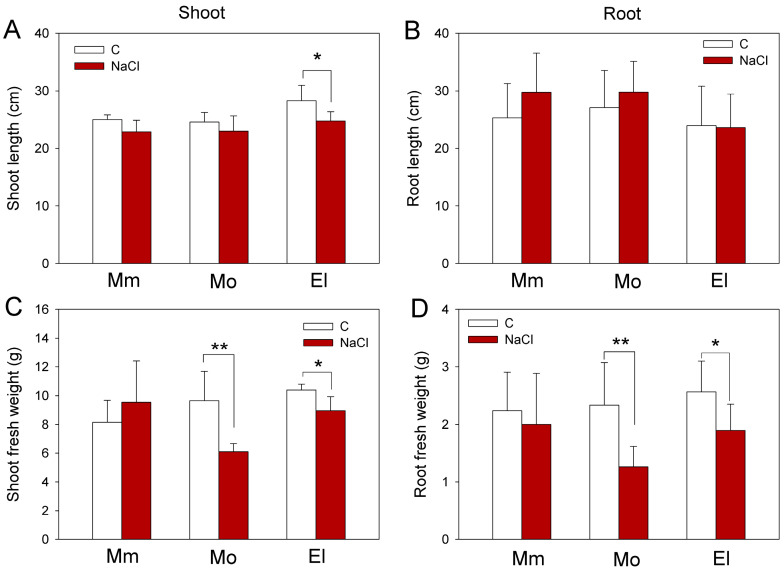
Growth of tomato plants under salt stress. Length and fresh weight of shoots (**A**,**C**) and roots (**B**,**D**) of five-week-old tomato plants measured under control conditions and after one week of 100 mM NaCl treatment. Mm—cv. Moneymaker, Mo—cv. Mobil, El—cv. Elán F1. Means ± SD. Significant differences according to the Student’s *t*-test (* *p* ≤ 0.05, ** *p* ≤ 0.01) are indicated.

The salt sensitivity of the tomato cultivars was evaluated by measuring the growth of the plants and by determining the H_2_O_2_ and lipid peroxide contents. Under control conditions, the measured growth parameters were similar in all three of the cultivars. After one week of 100 mM NaCl treatments, the shoot and root weights of the Mobil plants decreased more by the applied salt stress than the Moneymaker and Elán F1 plants, indicating that Mobil cultivar is the most sensitive to salt stress (Figure 1). The epinastic symptoms and the chlorotic older leaves of the salt-treated Mobil plants also support this result (Supplementary Figure S1).

**Figure 2:**
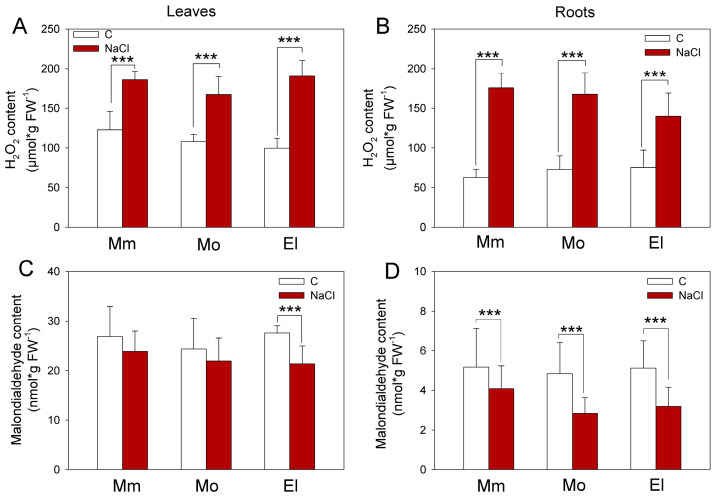
Hydrogen peroxide (H_2_O_2_) levels (**A**,**B**) and malondialdehyde content (**C**,**D**) of five-week-old tomato plants under control conditions and after one week of 100 mM NaCl treatment. Mm—cv. Moneymaker, Mo—cv. Mobil, El—cv. Elán F1. Means ± SD. Significant differences according to the Student’s *t*-test (*** *p* ≤ 0.001) are indicated.

Under control conditions, the H_2_O_2_ and the lipid peroxidation marker MDA contents in the leaves and roots were similar in the investigated cultivars (Figure 2). The salt treatment increased the H_2_O_2_ contents in all of the plants (Figure 2A,B) but, interestingly, they contained generally less MDA than the controls. Significant decreases were identified in the Elán F1 leaves and in the roots of all three of the cultivars (Figure 2C,D). Decreased MDA levels can be the result of the activation of antioxidant mechanisms. For example, several AtGSTU isoenzymes efficiently scavenge different reactive carbonyl species [59]; thus, these results suggest a cultivar-specific activation of the ROS-processing mechanisms, due to salt stress.

### 3.2. The High and/or Stress-Inducible, Non-Enzymatic Antioxidant Levels Promote an Effective Salt-Stress Response

**Figure 3:**
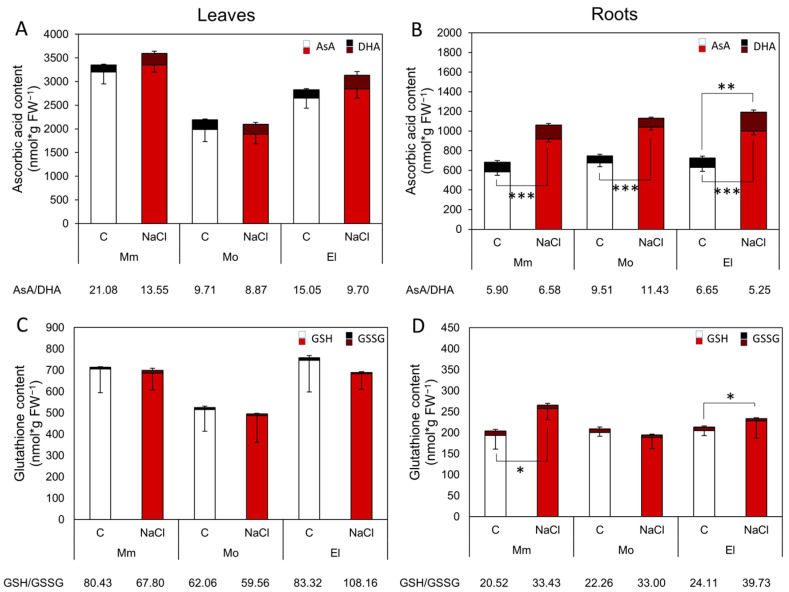
Ascorbic acid (reduced—AsA, oxidized—DHA; (**A**,**B**)) and glutathione (reduced—GSH, oxidized—GSSG; (**C**,**D**)) contents and their ratios in five-week-old tomato cultivars under control conditions and after one week of 100 mM NaCl treatment. Note the different vertical scales on the leaves (**A**,**C**) and roots (**B**,**D**). Mm—cv. Moneymaker, Mo—cv. Mobil, El—cv. Elán F1. Means ± SD. Significant differences according to the Student’s *t*-test (* *p* ≤ 0.05, ** *p* ≤ 0.01, *** *p* ≤ 0.001) are indicated.

To evaluate the non-enzymatic capacities of the tested tomato cultivars, reduced and oxidized ascorbate and glutathione contents were determined in the control and salt-treated plants. The AsA and GSH levels in the Mobil leaves were significantly lower than those in the Moneymaker and Elán F1 leaves under control conditions and after one-week-long salt stress (Figure 3A,C). Treatment with 100 mM NaCl elevated the AsA content in the roots of all three of the cultivars. The DHA content increased in the Elán F1 roots, thus, the AsA/DHA ratio was lowered; however, it was increased in the Moneymaker and Mobil plants after applying salt stress (Figure 3B). The GSH level was enhanced only in the Moneymaker roots after salt stress. The GSH/GSSG ratio increased in the roots of all of the tomato cultivars compared to the control plants and in the leaves of the Elán F1 plants. The calculated *E_GSH_* showed a more negative redox status after salt stress (Table 1), although no significant differences were recorded between the treatments. The Mobil plants had the least reduced *E_GSH_* values both in the leaves and roots of the investigated cultivars (Figure 3C,D and Table 1).

### 3.3. Higher Glutathione Reductase, Peroxidase, and Inducible Glutathione Transferase Activities Contribute to the Successful Salt-Stress Response of Tomatoes

**Figure 4:**
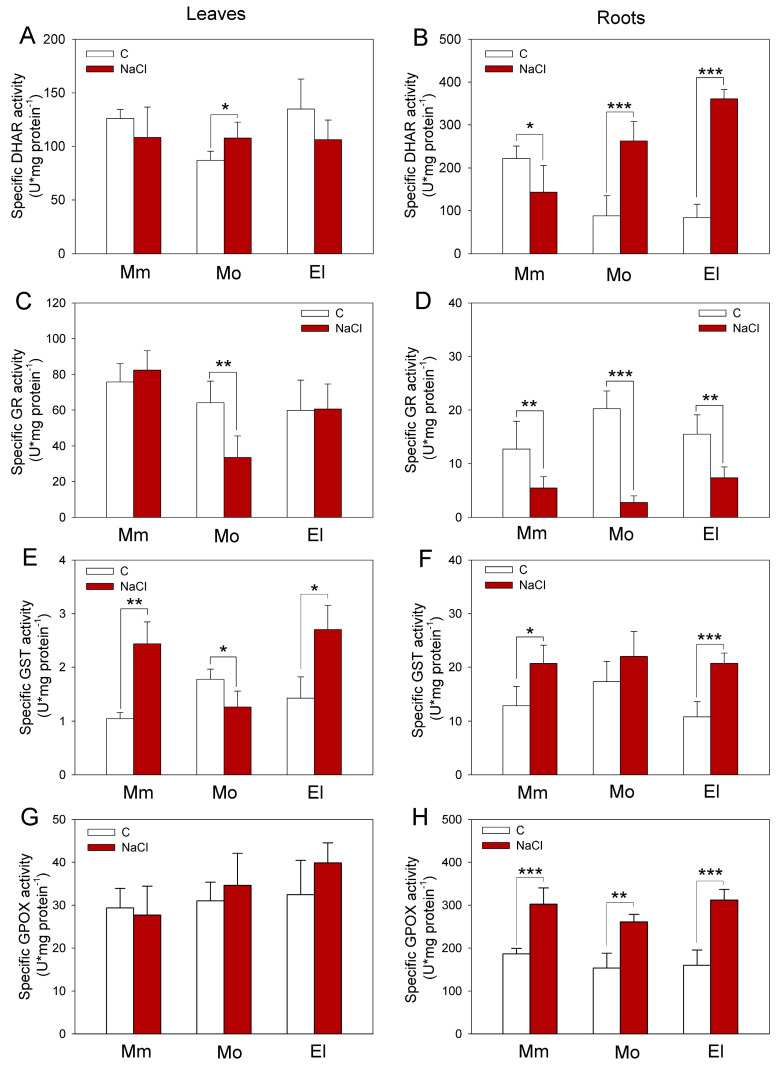
Specific dehydroascorbate reductase (DHAR), glutathione reductase (GR), transferase (GST), and peroxidase (GPOX) activities of tomato cultivars in five-week-old tomato leaves (**A**,**C**,**E**,**G**) and roots (**B**,**D**,**F**,**H**), respectively, under control conditions and after one week of 100 mM NaCl treatment. Mm—Moneymaker, Mo—Mobil, El—Elán F1. Means ± SD. Significant differences according to the Student’s *t*-test (* *p* ≤ 0.05, ** *p* ≤ 0.01, *** *p* ≤ 0.001) are indicated.

In order to characterize the antioxidant enzymes during salt stress, activities of GST, GR, DHAR, and GPOX were determined in the tested tomato cultivars. Under control conditions, the investigations revealed significantly higher GR activities in the Mobil roots, as well as slightly higher GST activities in the leaves compared to the Moneymaker and Elán F1 plants; however, its leaves showed the lowest DHAR activities (Figure 4). After seven days of salt stress, the DHAR activity was inhibited in the roots of the Moneymaker plants. In contrast, elevated DHAR activities were measured in the roots of both the Mobil (by 198.3%) and Elán F1 plants (by 329.8%) (Figure 4B). The one-week-long 100 mM NaCl treatment decreased the GR activities in the leaves of the Mobil plants and in the roots of all of the cultivars, but this decrease was most marked in the Mobil plants (Figure 4C,D). The GST activities were elevated in Moneymaker and Elán F1 leaves (by 160.1% and 91.7%, respectively) and roots (by 44.7% and 81.6%, respectively) after one week of 100 mM NaCl treatment. On the other hand, in the leaves of the Mobil plants, the GST activities decreased, while in the roots, only a slight increase was observed, due to the applied salt stress, compared to the control conditions (Figure 4E,F). The activity of GPOX remained unchanged in the leaves of all of the genotypes in response to stress but increased in the roots. The least change was observed in the Mobil cultivar (Figure 4G,H).

### 3.4. Expression Changes in the GR and Selected GST Genes in Salt-Treated Tomato Cultivars

The transcript levels of the 2 *GR* and 27 examined *GST* genes were detected by HT-qPCR from the leaves and roots of five-week-old tomato plants, whether or not they were subjected to the 100 mM NaCl treatment. The expression levels were generally the lowest in the Mobil plants, thus, the data for each gene from the Moneymaker and Elán F1 plants were normalized to those of Mobil. The transcript amount of the investigated genes under control conditions are shown in Supplementary Figure S2.

**Figure 5:**
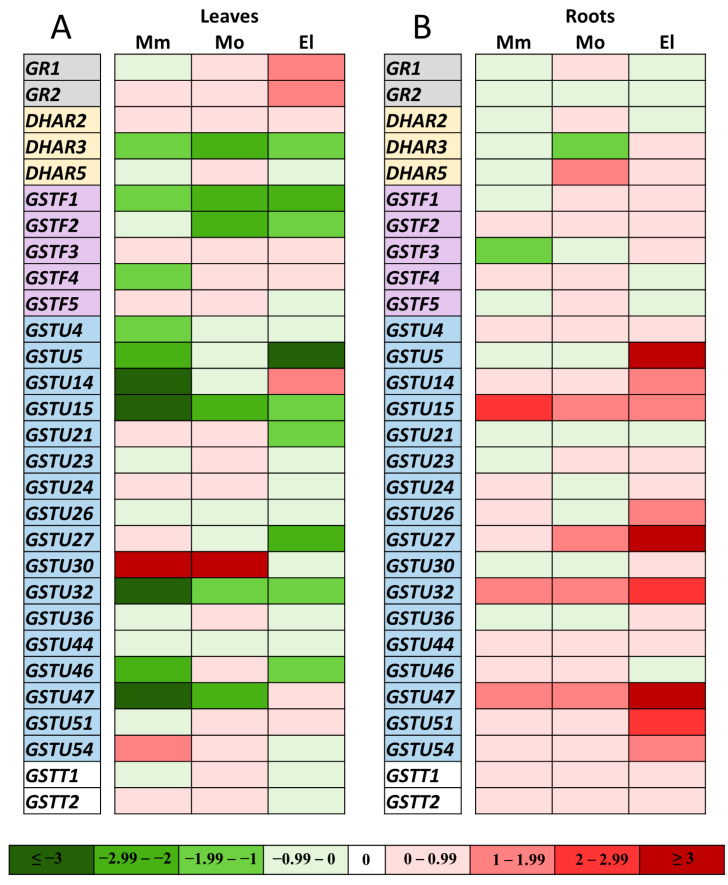
Heat map of the expression levels of 2 *Solanum lycopersicum* glutathione reductases (*GR1* and *GR2*) and 27 selected genes belonging to 4 GST classes (*DHAR*, *GSTF*, *GSTU*, *and GSTT*) determined in salt-treated, five-week-old Moneymaker (Mm), Mobil (Mo), and Elán F1 (El) tomato cultivars. The relative transcript amounts of the genes in the leaves (**A**) and roots (**B**) were determined by HT-qPCR after one week of 100 mM NaCl treatment. The expression of the genes was normalized first by the average of *actin2* and *elongation factor 1α* genes, and second by the average transcript amount of each gene in untreated cultivars. The log_2_ transformation of 2^−ΔΔCt^ data are presented. The green color represents repression, while the red color represents activation, as indicated on the color scale bar. The presented data are from two biological replicates.

To represent the effect of the 100 mM NaCl treatment on the transcription of the selected genes, the data were normalized to the untreated leaves and roots of each cultivar and for each gene. The results are displayed as a heat map (Figure 5). In the leaves, four genes (*DHAR3*, *GSTF1*, *GSTU15*, and *-32*) were downregulated in all of the cultivars after one week of salt stress. Beyond this, six genes showed downregulation in the Moneymaker leaves, two in the Mobil leaves, and five in the Elán F1 leaves, compared to the untreated leaves. At the same time, the expression of *GSTU30* was upregulated in the Moneymaker and Mobil plants, *GSTU54* was upregulated in the Moneymaker plants, and *GR1*, *GR2*, and *GSTU14* were upregulated in the Elán F1 plants (Figure 5A).

In the roots, *GSTU15*, *-32*, and *-47* were upregulated in all three of the cultivars after the salt treatments. In addition, the expression levels of six genes were upregulated in the Elán F1 plants and two in Mobil plants, by comparison with the untreated controls (Figure 5B). Downregulation was detected only at *GSTF3* in the Moneymaker plants and *DHAR3* in the Mobil plants after the applied salt stress.

### 3.5. Correlation Analysis of Gene Expressions

To reveal the relationship between the redox potential and the gene expression changes due to the NaCl treatment, Pearson’s correlation coefficients (R values) were determined. We used the direct (negative) *E_GSH_* and ΔCt values (Supplementary Table S4) for the calculation, in which a strong positive correlation means that, as the root tissues become more reduced, the expression level of a given gene increases, while in the case of a strong negative correlation, the transcript level of a specific gene increases as the root tissues become more oxidized. The correlation analysis resulted in considerable differences in the R values among the investigated cultivars and organs.

**Figure 6:**
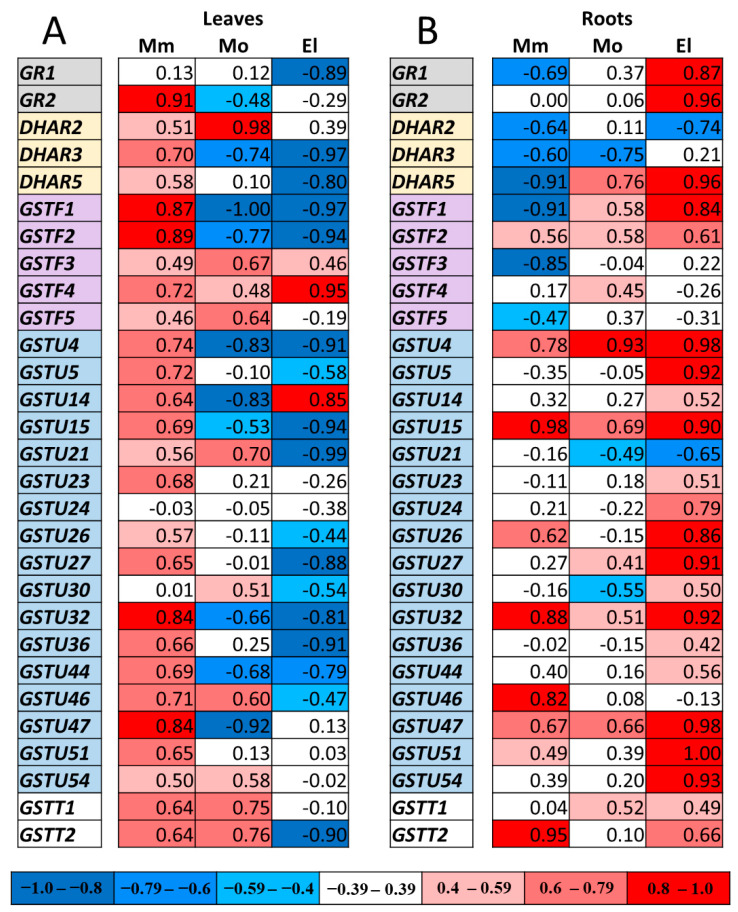
Correlation analysis between the glutathione redox potential (*E_GSH_*) and the expression of selected genes (ΔCt values), measured under different conditions in the leaves (**A**) and roots (**B**) of Moneymaker (Mm), Mobil (Mo), and Elán F1 (El) tomato plants. Positive correlations are highlighted in red and negative correlations are highlighted in blue.

Comparing the R values in the leaves of the cultivars revealed strong positive correlations between the changes in the expression of the selected genes and the *E_GSH_* values in almost all cases in the Moneymaker leaves, while in the case of the Elán F1 leaves, strong negative correlations were detected. For example, while *DHAR3*, *GSTU4*, *-5*, *-27*, and *-46* showed positive correlations with the redox potential in the Moneymaker leaves, a very strong negative correlation was found in the same set of genes in the Elán F1 leaves (Figure 6A). The positive correlations implicate the protective effect of the *GSTs* on the redox potential, while the negative correlations may indicate that the increasing damages enhanced the expression of the *GST* genes.

Regarding the roots, positive correlations were found between the expressions of *GSTF2*, -*U4*, -*U15*, -*U32*, -*U47*, and the *E_GSH_* in all of the cultivars (Figure 6B). Interestingly, the transcription of several *GSTs* related differently to the redox state in the roots rather than in the leaves. As an example, a strong positive correlation was found between the redox potential and the expression of the *GSTF1* gene in the Moneymaker leaves, while its transcript amount showed a strong negative correlation with the redox state in the roots (Figure 6A,B).

### 3.6. Involvement of the Cultivar-Specific, Stress-Responsive Transcription Factors in the Salt-Stress Response in Tomato Roots

**Figure 7:**
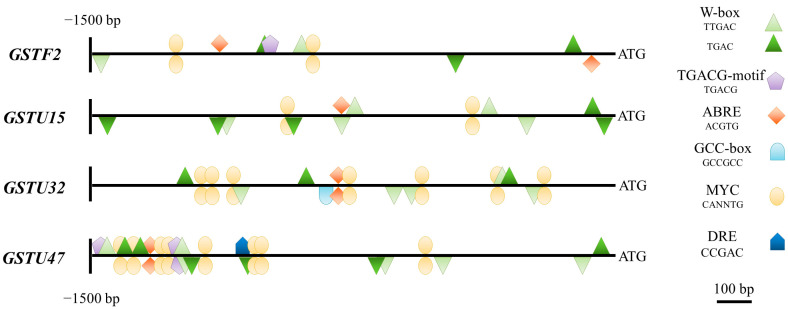
Predicted cis regulatory elements found in the upstream regulatory region of *GSTF2*, *GSTU15*, *GSTU32*, and *GSTU47* using the NewPLACE database (https://www.dna.affrc.go.jp/PLACE/?action=newplace, accessed on 25 February 2023).

Based on the results of the gene expression and correlation analysis, *GSTF2*, *GSTU15*, *-U32*, and *-U47* genes were selected for further in silico studies. These sequences were either upregulated (*GSTU15*, *-U32*, and *-U47*) or expressed at control levels (*GSTF2*) after one week of salt treatment in the roots of all of the cultivars. These showed a positive correlation with the further reduced redox potential (Figure 5 and Figure 6), indicating their important involvement in the primary defense mechanisms against salt stress. The in silico analysis of the 5′ regulatory region of *GSTU15*, *-U32*, and *-U47* genes revealed the presence of several W–box (TTGAC or TGAC), TGACG–motif (TGACG), and ABRE (ACGTG) elements in their promoter region (Figure 7). Additionally, *GSTU32* had a GCC-box (GCCGCC) and *GSTU47* had a DRE (CCGAC) element. In the promoter region of *GSTF2*, certain redox-responsive cis regulatory elements were also identified (Figure 7).

Accordingly, eight salt- and osmotic-stress-responsive TFs were selected for further gene expression studies, based on the data found in the literature and the results of the in silico promoter analysis (Supplementary Table S3). A comparison of their transcript levels in the leaves and roots of the three cultivars under control conditions showed low levels of differences (Supplementary Figure S3).

**Figure 8:**
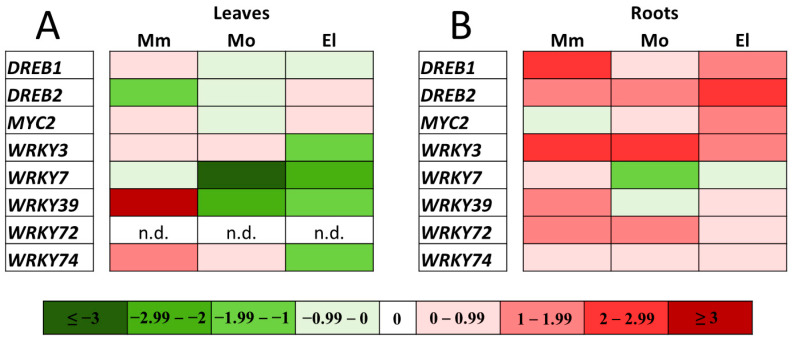
Heat map of the expression levels of eight *Solanum lycopersicum* transcription factor genes. The relative transcript levels of the selected genes were determined in five-week-old tomato (Moneymaker—Mm, Mobil—Mo, and Elán F1—El) leaves (**A**) and roots (**B**) by qPCR after one week of 100 mM NaCl treatment. For this, the expression values were normalized, firstly by the average expression of the *actin2* gene, and secondly by the average transcript levels of each gene from the untreated cultivars. The log_2_ transformation of the 2^−ΔΔCt^ data are presented as a heat map. The green color represents repression, while the red color represents activation, as indicated on the color scale bar. The presented data have been obtained from two biological replicates. n.d.—not detected.

However, significant changes occurred in their transcription after one week of salt treatment (Figure 8A,B). In the leaves, several of them were downregulated, compared to the untreated samples (*DREB2* in Moneymaker; *WRKY7* and *WRKY39* in Mobil and Elán F1; and *WRKY3* and *WRKY74* in the Elán F1 plants). Upregulation was detected only in the case of *WRKY39* and *WRKY74* in the Moneymaker leaves. In contrast, in the roots, most of the TFs were upregulated after one week of 100 mM NaCl treatment. *WRKY3* and *DREB2* were upregulated in all of the cultivars, *WRKY72* in the Moneymaker and Mobil cultivars, and *DREB1* both in the Moneymaker and Elán F1 cultivars, while *WRKY39* and *MYC2* were upregulated in the Moneymaker and Elán F1 cultivars. Downregulation was only observed in the *WRKY7* gene in the salt-stressed Mobil roots (Figure 8A,B).

**Figure 9:**
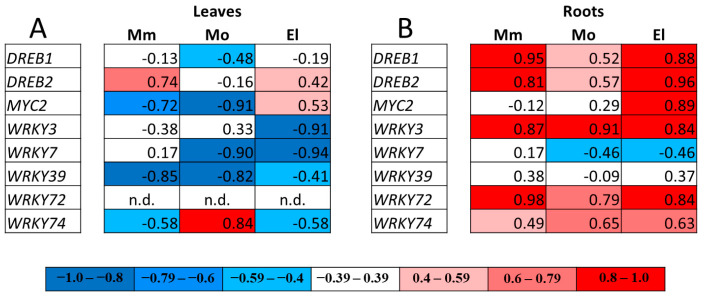
Correlation analysis on the basis of the glutathione redox potential (*E_GSH_*) and expression of selected genes (ΔCt values), measured under different conditions in the leaves (**A**) and roots (**B**) of Moneymaker (Mm), Mobil (Mo), and Elán F1 (El) tomato plants. Red color highlights the positive correlations and the blue color highlights the negative correlations. n.d.—not detected.

The correlation analysis performed between the redox potential and gene expression changes in the TFs showed differing results in the cultivars (Figure 9). In the leaves, negative correlations were found between the expression of *WRKY39* and the *E_GSH_* values in all of the cultivars, and that of several other TFs in one or two genotypes. In the roots, a strong positive correlation was found between the more negative GSH redox potential and the upregulation of *WRKY3* in all of the genotypes; furthermore, the strong positive correlations found in the case of *WRKY72*, *DREB1*, or *DREB2* and *E_GSH_* values in the Moneymaker and Elán F1 plants also suggest their importance in the salt-stress responses (Figure 9).

## 4. Discussion

### 4.1. Efficient Salt-Stress Responses Include Redox Status Changes and Activation of ROS-Processing Mechanisms to Adjust Plant Growth

It was reported that the growth parameters of the salt-tolerant wild relatives of the tomato (like *Solanum pennellii*, *Solanum galapagense*, *Solanum cheesmaniae*, and *Solanum chilense*) are generally behind the cultivated variants under optimal conditions; however, these can surpass the domesticated tomatoes under high levels of salt stress [60]. An effective salt response was characterized, not only with restored shoot growth, but also with increased growth and higher root biomass accumulation, under severe salinity stress [61]. In our experiments, a comparison of the salt-stress responses of three tomato cultivars revealed that the Moneymaker cultivar preserved better its growth after one week of salt stress, but significant growth inhibition was detected in the Mobil plants (Figure 1), indicating that the Moneymaker cultivar is the most salt tolerant, while Mobil is the most sensitive cultivar. Although the Moneymaker and Elán F1 cultivars could maintain their growth during the seven days of the 100 mM NaCl treatments, higher H_2_O_2_ levels were measured in all of the cultivars after salt stress, which was not associated with elevated MDA contents (Figure 2), suggesting that the elevated ROS may serve other, presumably signaling, purposes.

It is well documented that endogenous ROS levels, changes in the redox status, non-enzymatic antioxidants, and enzymes serve as key signaling agents; moreover, the enhancement of GSH and AsA pools promotes adaptation to various types of stresses [31,62,63,64]. Using the redox-sensitive roGFP redox probe to analyze the in vivo changes in *A. thaliana* roots after NaCl treatment, Jiang et al. have shown that there is a rapid change in the redox profile, but the redox state can be restored within 24 h [65]. Changes in the redox state, even a few mV shift, implies modulation of cellular signaling and alterations in gene expression profiles, including reprogramming the expression of a set of hormone- and defense-related genes [36,65,66]. The GSH redox-state-dependent transcriptional changes in *GST* genes were reported either in plants with low GSH levels [36] or after applying different stress treatments [19,20,21,66]. However, other than the ROS-processing role of GSTs, their involvement in altering metabolic and growth responses should be elucidated.

### 4.2. The Upregulation of Several Genes of GSH-Related Enzymes Correlates with the More Reduced GSH Redox Status

The multigenic superfamily of GSTs is one of nature’s most versatile enzymes. Besides their well-known reaction, namely, the conjugation of GSH to electrophilic compounds, numerous GSTs act as GSH-dependent peroxidases by reducing organic hydroperoxides [3,67]. The earlier results obtained from salt-treated *Atgstu19*, *Atgstu24*, *Atgstf8*, and *Atgstf9* mutants have shown that *A. thaliana* GST isoenzymes have different roles in determining the level of specific ROS species and participate in the establishment of the root redox status [19,20,40]. In tomato roots, one day of 150 mM NaCl treatment increased the expression of the *GST* genes belonging to the tau, phi, and theta groups, along with GST and GPOX activities [21]. The upregulation of *GST* genes under several stress conditions was related to the elevated total GST enzyme activity [11]. Our results showed that GST activities were elevated in the Moneymaker and Elán F1 cultivars after one week of salt stress, but not in the salt-sensitive Mobil variety (Figure 4). In our earlier study, elevated GST and GPOX activities were detected in the untreated roots of Moneymaker compared to Ailsa Craig cultivars. Moreover, the importance of the redox status and its genotype-specific significance in stress responses were indicated [21]. In this study, differences were found in the pool and redox status of the AsA and GSH of the three tomato cultivars, even under control conditions, which was augmented by salt stress. Lower AsA and GSH levels were measured in the leaves of the Mobil plants, and, compared to the other cultivars, the lowest AsA/DHA and GSH/GSSG ratios and more positive *E_GSH_* values were observed in the leaves. While in the Moneymaker cultivar, the accumulation of GSH contributed to the maintenance of redox homeostasis, in the Mobil and Elán F1 plants, the elevated DHAR activities could support the protection of the GSH pool (Figure 3 and Figure 4). DHARs are also involved in balancing redox homeostasis in plants, as they catalyze the GSH-dependent reduction of DHA to AsA [1,68,69]. Moreover, increased AsA levels and the overexpression of DHARs were reported to promote a tolerance to multiple stresses [70,71,72,73,74,75,76].

The main basis of the ROS/redox regulation is primarily based on the redox state of Cys residues, and among the main molecules affected are thioredoxin (TRX) and GSH [77]. Regarding GST proteins, DHARs and GSTLs with Cys residues in their active site can be modified by GSH to form mixed disulfides, while GSTZ1, GSTF7, and GSTU19 were identified as S-glutathionylated (thiolated) proteins in *Arabidopsis* [1,78]. Several GST isoenzymes were annotated as phosphopeptides, while, in others, methionine undergoes sulfur oxidation or cysteins form reversible intramolecular disulfide bonds, which affect the activity of the GSTs [79,80,81].

Previously, we found that a remarkably high number of *GST* genes were expressed at a higher level in the untreated roots of Moneymaker plants compared to the less stress-resistant Ailsa Craig cultivar. Furthermore, the transcript levels of several *GST* genes and other genes coding GSH-related enzymes showed a positive correlation with elevated GSH levels and a further reduced *E_GSH_* after 24 h of the applied treatments, especially in Moneymaker plants [21]. In the present experiments, significant but diverse correlations were found between the expression of the investigated *GSTs* and the calculated GSH redox potentials, depending on the organs and genotypes. Positive correlations might indicate that the increased expression of those genes has a protective effect on the tissue redox status. The members of the investigated four GST classes are involved in the detoxification of stress metabolites, thus, our results strengthen the idea that several GSTs are part of the well-ordered systematic antioxidant response. In the leaves, 22 *GST* genes were expressed at a higher level in the Moneymaker cultivars compared to the Mobil under control conditions (Supplementary Figure S2A). Although fewer differences were found in the expression of the investigated genes in the roots of the untreated tomato cultivars than in their leaves, our results show that the tolerant genotype possess more efficient protective mechanisms, which might prevent oxidative damages in the Moneymaker cultivar. In the case of other genotypes, negative correlations were found between the expression of several *SlGSTs* and the redox potential (Figure 6). This suggests that, in such a case, the defense mechanisms might be activated when the oxidative damages were rather high (the tolerance mechanisms positively correlate with oxidative damage).

### 4.3. Strong and Cultivar-Specific Correlations Were Found in the Changes in Redox Potential and the Expression of TF Genes

In plants, transcriptional responses are regulated either by stress- or ROS-derived changes in the signal transduction mechanisms or by direct or indirect ROS-induced redox regulation [82]. The 5′ regulatory regions (1500 bp upstream from the start codon) of the investigated *GST* sequences found on the SGN (http://solgenomics.net, accessed on 1 May 2022) homepage were screened for the presence of oxidative stress- and redox-related cis regulatory elements, in particular, using the NewPLACE database. A number of redox-responsive cis elements were found in the regulatory regions of the *GST* genes, such as W–box (TTGAC), TGACG–motif (TGACG), ABRE (ACGTG), and GCC-box (GCCGCC) motifs, potential binding sites for AP2, bZIP, and WRKY-type transcription factors (Supplementary Table S1). Investigating the expression of eight stress-responsive TFs (*DREB1*, *DREB2*, *MYC2*, *WRKY3*, *-7*, -*39*, -*72*, and -*74*) using qPCR revealed potential regulatory differences between these cultivars. In the leaves, the upregulation of the TF genes due to the applied salt treatment (*WRKY74* and *WRKY39*) was detected only in the Moneymaker plants. In the roots, upregulated expression levels were found in the case of five, four, and three TFs in the Moneymaker, Elán F1, and Mobil cultivars, respectively, after salt stress, from which we highlight here the TFs belonging to the WRKY and DREB families.

In tomatoes, 81 *WRKY* genes were annotated, some of which have already been reported to be stress-induced [83]. Among several others, *SlWRKY8*, *SlWRKY39*, *SlWRKY72*, and *SlWRKY74* showed induction during salt, drought, and biotic stresses [83,84]. SlWRKY39 is a positive regulator in tomatoes’ response to biotic and abiotic stresses; moreover, the overexpression of this TF activates the expression of pathogenesis- and environmental-stress-related genes [85]. WRKY72 has been described to play an important role in conferring drought tolerance in tomato plants [86]. DREB1 and DREB2 are dehydration-responsive element-binding TFs, which affect the development of vegetative and reproductive organs, participate in abscisic acid signaling and in the drought-stress response, and, furthermore, mediate salt-stress tolerance in tomato plants [87,88,89]. The strong positive correlations between the expression of their genes and the redox potential in tomato roots also indicate their involvement in the maintenance of the redox status of tissues.

**Figure 10:**
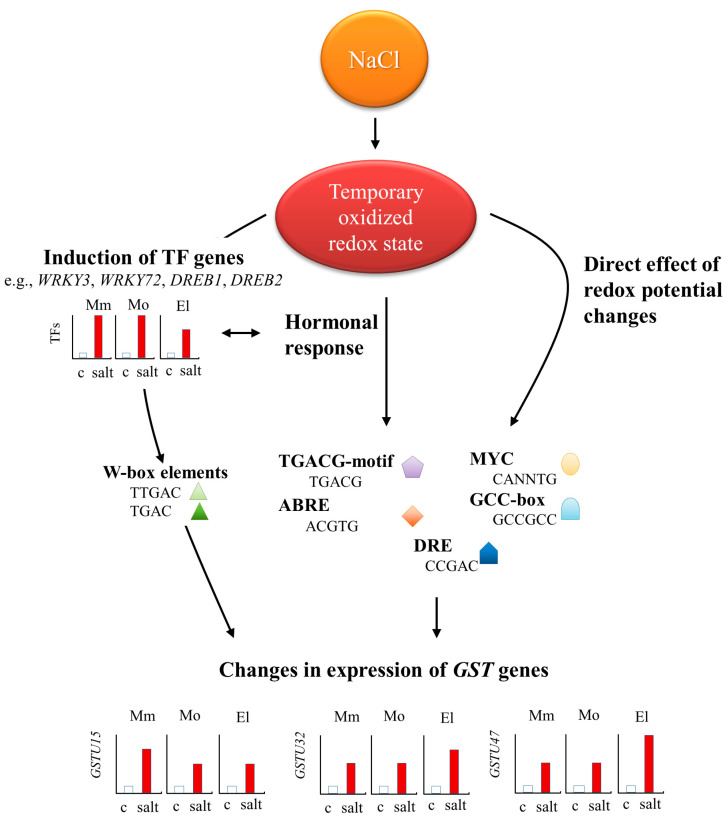
Schematic model summarizing the potential regulators of *GST* gene expression after one week 100 mM NaCl treatments in tomato plants, based on our results and data found in the literature [90,91]. The salt treatment supposedly disturbed the redox homeostasis, which led to a temporary oxidized redox state within the cells. These changes in the redox potential may have had a direct effect on the expression of *GSTs*, but also led to the induction of the TF genes, such as *WRKY3*, *WRKY72*, *DREB1*, and *DREB2*. Presumably, the expression of these genes was also influenced by hormonal changes or, for example, 5′ cis regulatory elements could also induce the expression of *GSTU15*, *GSTU32*, and *GSTU47* genes in all investigated cultivars. Abbreviations: Moneymaker—Mm; Mobil—Mo; Elán F1—El; control—C; salt—one week of 100 mM NaCl treatment.

A schematic model of the regulatory mechanisms involved in the induced expression of tomato *GSTs* is represented in Figure 10. A relationship was found between the expression of the *WRKY3* gene and several plant hormones, such as salicylic acid, cytokinin, indole-3-butyric acid, and jasmonoyl-isoleucine [90,91]. Based on our findings, *WRKY3*, *WRKY72*, *DREB1*, and *DREB2*, together with other stress-related hormones and redox-state-dependent signaling events, upregulate the expression of *GSTU15*, *GSTU32*, and *GSTU47*, which may be partially responsible for the increased GST activity, leading to more successful stress tolerance.

## 5. Conclusions

Our results indicate that the Moneymaker and Elán F1 cultivars were able to restore their growth during one-week-long salt stress, indicating a more efficient stress response compared to the Mobil cultivar, the growth of which decreased after the stress treatment. These results indicate that lower GSH and AsA levels, more positive glutathione redox potential, and decreased GR and GST activities can negatively influence the growth of Mobil plants during salt stress. Since the Moneymaker and Elán F1 plants had higher GR and inducible GST activities, along with elevated non-enzymatic antioxidant levels, the combination of these factors is likely to have contributed to the salt tolerance of these plants. Analyses between the redox potential values and the gene expression data revealed several strong and cultivar-specific correlations, highlighting the importance of GSTs in the complex stress responses of tomato plants. Transcription factors *WRKY3*, *WRKY72*, *DREB1*, and *DREB2* can be involved in *GST* gene induction, which may be partly responsible for increased GST activity, leading to more efficient stress tolerance in tomato plants. These results confirm how a specific set of *GSTs* show a genotype-specific spatiotemporal expression pattern in the salt response of tomato cultivars, and these alterations in their expression determine their flexibility under stress conditions.

## Figures and Tables

**Table 1:** Glutathione redox potential (*E_GSH_*) of hydroponically grown five-week-old *Solanum lycopersicum* Moneymaker (Mm), Mobil (Mo), and Elán F1 (El) plants after one week of 100 mM NaCl treatment. No significant differences were detected according to the Student’s *t*-test.

*E_GSH_* (mV)				
	Leaves		Roots	
	Control	NaCl	Control	NaCl
Mm	−321.4 ± 4.5	−319.9 ± 3.0	−286.7 ± 10.7	−292.4 ± 8.4
Mo	−312.7 ± 4.7	−312.9 ± 5.7	−285.5 ± 11.1	−290.1 ± 7.7
El	−323.9 ± 5.2	−326.0 ± 8.8	−287.4 ± 6.7	−292.3 ± 9.0

## Data Availability

The data presented in this study are available in the article and supplementary materials or are available upon request from the corresponding author.

## Supplementary Materials

The following supporting information can be downloaded at: https://www.mdpi.com/article/10.3390/antiox12091682/s1, Figure S1: Image of a five-week-old tomato plant under control conditions and after one week of 100 mM NaCl treatment; Figure S2: Expression of *GRs* and *GSTs* under control conditions; Figure S3: Expression of TFs in control plants; Table S1: Number of redox-related cis regulator sequences in *GSTs*; Table S2: Primer pairs used in this study; Table S3: Selection of the TFs; Table S4: ∆Ct values used in correlation analysis. References [92,93] are cited in the supplementary materials.

## References

[1] Dixon D.P., Davis B.G., Edwards R. (2002). Functional divergence in the glutathione transferase superfamily in plants. J. Biol. Chem..

[2] Labrou N.E., Papageorgiou A.C., Pavli O., Flemetakis E. (2015). Plant GSTome: Structure and functional role in xenome network and plant stress response. Curr. Opin. Biotechnol..

[3] Hernández Estévez I., Rodríguez Hernández M. (2020). Plant glutathione S-transferases: An overview. Plant Gene.

[4] Frear D.S., Swanson H.R. (1970). Biosynthesis of S-(4-ethylamino-6-isopropylamino- 2-S-triazino) glutathione: Partial purification and properties of a glutathione S-transferase from corn. Phytochemistry.

[5] Edwards R., Dixon D.P. (2005). Plant glutathione transferases. Methods Enzymol..

[6] Dixon D.P., Skipsey M., Edwards R. (2010). Roles for glutathione transferases in Plant Secondary Metabolism. Phytochemistry.

[7] Cummins I., Dixon D.P., Freitag-Pohl S., Skipsey M., Edwards R. (2011). Multiple roles for plant glutathione transferases in xenobiotic detoxification. Drug Metab. Rev..

[8] Wagner U., Edwards R., Dixon D.P., Mauch F. (2002). Probing the diversity of the Arabidopsis glutathione S-transferase gene family. Plant Mol. Biol..

[9] Sappl P.G., Oñate-Sánchez L., Singh K.B., Millar A.H. (2004). Proteomic analysis of glutathione S-transferases of *Arabidopsis thaliana* reveals differential salicylic acid-induced expression of the plant-specific phi and tau classes. Plant Mol. Biol..

[10] Sappl P.G., Carroll A.J., Clifton R., Lister R., Whelan J., Harvey Millar A., Singh K.B. (2009). The Arabidopsis glutathione transferase gene family displays complex stress regulation and co-silencing multiple genes results in altered metabolic sensitivity to oxidative stress. Plant J..

[11] Islam S., Rahman I.A., Islam T., Ghosh A. (2017). Genome-wide identification and expression analysis of glutathione S-transferase gene family in tomato: Gaining an insight to their physiological and stress-specific roles. PLoS ONE.

[12] Ding F., Wang C., Zhang S., Wang M. (2022). A jasmonate-responsive glutathione S-transferase gene *SlGSTU24* mitigates cold-induced oxidative stress in tomato plants. Sci. Hortic..

[13] Otulak-Kozieł K., Kozieł E., Horváth E., Csiszár J. (2022). AtGSTU19 and AtGSTU24 as moderators of the response of *Arabidopsis thaliana* to *Turnip mosaic* virus. Int. J. Mol. Sci..

[14] Chen J.-H., Jiang H.-W., Hsieh E.-J., Chen H.-Y., Chien C.-T., Hsieh H.-L., Lin T.-P. (2012). Drought and salt stress tolerance of an arabidopsis glutathione *s*-transferase U17 knockout mutant are attributed to the combined effect of glutathione and abscisic acid. Plant Physiol..

[15] Csiszár J., Horváth E., Váry Z., Gallé Á., Bela K., Brunner S., Tari I. (2014). Glutathione transferase Supergene family in Tomato: Salt stress-regulated expression of representative genes from distinct GST classes in plants primed with salicylic acid. Plant Physiol. Biochem..

[16] Tari I., Csiszár J., Horváth E., Poór P., Takács Z., Szepesi Á. (2015). The alleviation of the adverse effects of salt stress in the tomato plant by salicylic acid shows a time- and organ-specific antioxidant response. Acta Biol. Cracoviensia Ser. Bot..

[17] Xu J., Xing X.-J., Tian Y.-S., Peng R.-H., Xue Y., Zhao W., Yao Q.-H. (2015). Transgenic *Arabidopsis* plants expressing tomato glutathione S-transferase showed enhanced resistance to salt and drought stress. PLoS ONE.

[18] Horváth E., Brunner S., Bela K., Papdi C., Szabados L., Tari I., Csiszár J. (2015). Exogenous salicylic acid-triggered changes in the glutathione transferases and peroxidases are key factors in the successful salt stress acclimation of *Arabidopsis thaliana*. Funct. Plant Biol..

[19] Horváth E., Bela K., Holinka B., Riyazuddin R., Gallé Á., Hajnal Á., Hurton Á., Fehér A., Csiszár J. (2019). The *Arabidopsis* glutathione transferases, ATGSTF8 and ATGSTU19 are involved in the maintenance of root redox homeostasis affecting meristem size and salt stress sensitivity. Plant Sci..

[20] Horváth E., Bela K., Gallé Á., Riyazuddin R., Csomor G., Csenki D., Csiszár J. (2020). Compensation of mutation in *Arabidopsis glutathione transferase* (*AtGSTU*) genes under control or salt stress conditions. Int. J. Mol. Sci..

[21] Gallé Á., Bela K., Hajnal Á., Faragó N., Horváth E., Horváth M., Puskás L., Csiszár J. (2021). Crosstalk between the redox signalling and the detoxification: GSTs under redox control?. Plant Physiol. Biochem..

[22] van Zelm E., Zhang Y., Testerink C. (2020). Salt tolerance mechanisms of plants. Annu. Rev. Plant Biol..

[23] Munns R., Tester M. (2008). Mechanisms of salinity tolerance. Annu. Rev. Plant Biol..

[24] Zörb C., Geilfus C.M., Dietz K.J. (2018). Salinity and crop yield. Plant Biol..

[25] Zhu J.-K. (2001). Plant Salt Tolerance. Trends Plant Sci..

[26] Liang W., Ma X., Wan P., Liu L. (2018). Plant Salt-tolerance mechanism: A Review. Biochem. Biophys. Res. Commun..

[27] Hasanuzzaman M., Bhuyan M.H., Parvin K., Bhuiyan T.F., Anee T.I., Nahar K., Hossen M.S., Zulfiqar F., Alam M.M., Fujita M. (2020). Regulation of ROS metabolism in plants under environmental stress: A review of recent experimental evidence. Int. J. Mol. Sci..

[28] Sies H., Belousov V.V., Chandel N.S., Davies M.J., Jones D.P., Mann G.E., Murphy M.P., Yamamoto M., Winterbourn C. (2022). Defining roles of specific reactive oxygen species (ROS) in cell biology and physiology. Nat. Rev. Mol. Cell Biol..

[29] Štolfa I., Špoljarić Maronić D., Žuna Pfeiffer T., Lončarić Z., Corpas F.J., Gupta D.K., Palma José M. (2016). Glutathione and related enzymes in response to abiotic stress. Redox State as a Central Regulator of Plant-Cell Stress Responses.

[30] Potters G., Horemans N., Jansen M.A.K. (2010). The cellular redox state in plant stress biology—A charging concept. Plant Physiol. Biochem..

[31] Foyer C.H., Noctor G. (2011). Ascorbate and glutathione: The heart of the redox hub. Plant Physiol..

[32] Gill S.S., Anjum N.A., Hasanuzzaman M., Gill R., Trivedi D.K., Ahmad I., Pereira E., Tuteja N. (2013). Glutathione and glutathione reductase: A boon in disguise for plant abiotic stress defense operations. Plant Physiol. Biochem..

[33] Noctor G., Mhamdi A., Chaouch S., Han Y., Neukermans J., Marquez-Garcia B., Queval G., Foyer C.H. (2011). Glutathione in plants: An integrated overview. Plant Cell Environ..

[34] Cheng J.C., Seeley K.A., Sung Z.R. (1995). RML1 and RML2, Arabidopsis genes required for cell proliferation at the root tip. Plant Physiol..

[35] Vernoux T., Wilson R.C., Seeley K.A., Reichheld J.-P., Muroy S., Brown S., Maughan S.C., Cobbett C.S., Montagu M.V., Inze D. (2000). The root meristemless1/cadmium sensitive2 gene defines a glutathione-dependent pathway involved in initiation and maintenance of cell division during Postembryonic Root Development. Plant Cell.

[36] Schnaubelt D., Queval G., Dong Y., Diaz-Vivancos P., Makgopa M.E., Howell G., De Simone A., Bai J., Foyer C.H. (2014). Low glutathione regulates gene expression and the redox potentials of the nucleus and cytosol in *Arabidopsis thaliana*. Plant Cell Environ..

[37] Aller I., Rouhier N., Meyer A.J. (2013). Development of roGFP2-derived redox probes for measurement of the glutathione redox potential in the cytosol of severely glutathione-deficient *rml1* seedlings. Front. Plant Sci..

[38] Csiszár J., Brunner S., Horváth E., Bela K., Ködmön P., Riyazuddin R., Gallé Á., Hurton Á., Papdi C., Szabados L. (2018). Exogenously applied salicylic acid maintains redox homeostasis in salt-stressed *Arabidopsis gr1* mutants expressing cytosolic roGFP1. Plant Growth Regul..

[39] Rahantaniaina M.-S., Tuzet A., Mhamdi A., Noctor G. (2013). Missing links in understanding redox signaling via thiol/disulfide modulation: How is glutathione oxidized in plants?. Front. Plant Sci..

[40] Horváth E., Bela K., Papdi C., Gallé Á., Szabados L., Tari I., Csiszár J. (2015). The role of *Arabidopsis glutathione transferase F9* gene under oxidative stress in seedlings. Acta Biol. Hung..

[41] Munyampundu J.-P., Xu Y.-P., Cai X.-Z. (2016). Phi class of glutathione S-transferase gene superfamily widely exists in nonplant taxonomic groups. Evol. Bioinform..

[42] Lallement P.-A., Brouwer B., Keech O., Hecker A., Rouhier N. (2014). The still mysterious roles of cysteine-containing glutathione transferases in plants. Front. Pharmacol..

[43] Liu Y.-J., Han X.-M., Ren L.-L., Yang H.-L., Zeng Q.-Y. (2013). Functional divergence of the glutathione *s*-transferase supergene family in *Physcomitrella patens* reveals complex patterns of large gene family evolution in land plants. Plant Physiol..

[44] Kampranis S.C., Damianova R., Atallah M., Toby G., Kondi G., Tsichlis P.N., Makris A.M. (2000). A novel plant glutathione S-transferase/peroxidase suppresses Bax lethality in yeast. J. Biol. Chem..

[45] Kilili K.G., Atanassova N., Vardanyan A., Clatot N., Al-Sabarna K., Kanellopoulos P.N., Makris A.M., Kampranis S.C. (2004). Differential roles of tau class glutathione S-transferases in oxidative stress. J. Biol. Chem..

[46] Sun W., Xu X., Zhu H., Liu A., Liu L., Li J., Hua X. (2010). Comparative transcriptomic profiling of a salt-tolerant wild tomato species and a salt-sensitive tomato cultivar. Plant Cell Physiol..

[47] Mittova V., Theodoulou F.L., Kiddle G., Gómez L., Volokita M., Tal M., Foyer C.H., Guy M. (2003). Coordinate induction of glutathione biosynthesis and glutathione-metabolizing enzymes is correlated with salt tolerance in tomato. FEBS Lett..

[48] Mátai A., Hideg É. (2017). A comparison of colorimetric assays detecting hydrogen peroxide in leaf extracts. Anal. Methods.

[49] Ederli L., Pasqualini S., Batini P., Antonielli M. (1997). Photoinhibition and oxidative stress: Effects on xanthophyll cycle, scavenger enzymes and abscisic acid content in tobacco plants. J. Plant Physiol..

[50] Bela K., Riyazuddin R., Horváth E., Hurton Á., Gallé Á., Takács Z., Zsigmond L., Szabados L., Tari I., Csiszár J. (2018). Comprehensive analysis of antioxidant mechanisms in *Arabidopsis* glutathione peroxidase-like mutants under salt- and osmotic stress reveals organ-specific significance of the AtGPXL’s activities. Environ. Exp. Bot..

[51] Gillespie K.M., Ainsworth E.A. (2007). Measurement of reduced, oxidized and total ascorbate content in plants. Nat. Protoc..

[52] Schafer F.Q., Buettner G.R. (2001). Redox environment of the cell as viewed through the redox state of the glutathione disulfide/glutathione couple. Free Radic. Biol. Med..

[53] Bradford M.M. (1976). A rapid and sensitive method for the quantitation of microgram quantities of protein utilizing the principle of protein-dye binding. Anal. Biochem..

[54] Smith I.K., Vierheller T.L., Thorne C.A. (1988). Assay of glutathione reductase in crude tissue homogenates using 5,5′-dithiobis(2-nitrobenzoic acid). Anal. Biochem..

[55] Habig W.H., Pabst M.J., Jakoby W.B. (1974). Glutathione S-transferases: The first enzymatic step in mercapturic acid formation. J. Biol. Chem..

[56] Awasthi Y.C., Beutler E., Srivastava S.K. (1975). Purification and properties of human erythrocyte glutathione peroxidase. J. Biol. Chem..

[57] Livak K.J., Schmittgen T.D. (2001). Analysis of relative gene expression data using real-time quantitative PCR and the 2^−ΔΔCT^ method. Methods.

[58] Higo K. (1999). Place: A database of plant cis-acting regulatory DNA elements. Nucleic Acids Res..

[59] Mano J., Kanameda S., Kuramitsu R., Matsuura N., Yamauchi Y. (2019). Detoxification of reactive carbonyl species by glutathione transferase tau isozymes. Front. Plant Sci..

[60] Bonarota M.-S., Kosma D.K., Barrios-Masias F.H. (2021). Salt tolerance mechanisms in the *lycopersicon* clade and their trade-offs. AoB Plants.

[61] Lovelli S., Scopa A., Perniola M., Di Tommaso T., Sofo A. (2012). Abscisic acid root and leaf concentration in relation to biomass partitioning in salinized tomato plants. J. Plant Physiol..

[62] Antoniou C., Savvides A., Christou A., Fotopoulos V. (2016). Unravelling chemical priming machinery in plants: The role of reactive oxygen–nitrogen–sulfur species in abiotic stress tolerance enhancement. Curr. Opin. Plant Biol..

[63] Foyer C.H., Noctor G. (2020). Redox homeostasis and signaling in a higher-CO_2_ world. Annu. Rev. Plant Biol..

[64] Considine M.J., Foyer C.H. (2021). Stress effects on the reactive oxygen species-dependent regulation of plant growth and development. J. Exp. Bot..

[65] Jiang K., Moe-Lange J., Hennet L., Feldman L.J. (2016). Salt stress affects the redox status of Arabidopsis root meristems. Front. Plant Sci..

[66] Ugalde J.M., Lamig L., Herrera-Vásquez A., Fuchs P., Homagk M., Kopriva S., Müller-Schüssele S.J., Holuigue L., Meyer A.J. (2021). A dual role for glutathione transferase U7 in plant growth and protection from methyl viologen-induced oxidative stress. Plant Physiol..

[67] Sylvestre-Gonon E., Law S.R., Schwartz M., Robe K., Keech O., Didierjean C., Dubos C., Rouhier N., Hecker A. (2019). Functional, structural and biochemical features of plant serinyl-glutathione transferases. Front. Plant Sci..

[68] Foyer C.H., Halliwell B. (1976). The presence of glutathione and glutathione reductase in chloroplasts: A proposed role in ascorbic acid metabolism. Planta.

[69] Noctor G., Arisi A.-C.M., Jouanin L., Kunert K.J., Rennenberg H., Foyer C.H. (1998). Glutathione: Biosynthesis, metabolism and relationship to stress tolerance explored in transformed plants. J. Exp. Bot..

[70] Eltayeb A.E., Kawano N., Badawi G.H., Kaminaka H., Sanekata T., Morishima I., Shibahara T., Inanaga S., Tanaka K. (2006). Enhanced tolerance to ozone and drought stresses in transgenic tobacco overexpressing dehydroascorbate reductase in cytosol. Physiol. Plant..

[71] Yin L., Wang S., Eltayeb A.E., Uddin M.I., Yamamoto Y., Tsuji W., Takeuchi Y., Tanaka K. (2009). Overexpression of dehydroascorbate reductase, but not monodehydroascorbate reductase, confers tolerance to aluminum stress in transgenic tobacco. Planta.

[72] Wang Z., Xiao Y., Chen W., Tang K., Zhang L. (2010). Increased vitamin C content accompanied by an enhanced recycling pathway confers oxidative stress tolerance in *Arabidopsis*. J. Integr. Plant Biol..

[73] Li Q., Li Y., Li C., Yu X. (2012). Enhanced ascorbic acid accumulation through overexpression of dehydroascorbate reductase confers tolerance to methyl viologen and salt stresses in tomato. Czech J. Genet. Plant Breed..

[74] Shin S.-Y., Kim M.-H., Kim Y.-H., Park H.-M., Yoon H.-S. (2013). Co-expression of monodehydroascorbate reductase and dehydroascorbate reductase from *Brassica rapa* effectively confers tolerance to freezing-induced oxidative stress. Mol. Cells.

[75] Kim Y.S., Kim I.S., Shin S.Y., Park T.H., Park H.M., Kim Y.H., Lee G.S., Kang H.G., Lee S.H., Yoon H.S. (2014). Overexpression of dehydroascorbate reductase confers enhanced tolerance to salt stress in rice plants (*Oryza sativa* L. *japonica*). J. Agron. Crop Sci..

[76] Kim Y.-S., Park S.-I., Kim J.-J., Shin S.-Y., Kwak S.-S., Lee C.-H., Park H.-M., Kim Y.-H., Kim I.-S., Yoon H.-S. (2022). Over-expression of dehydroascorbate reductase improves salt tolerance, environmental adaptability and productivity in *Oryza sativa*. Antioxidants.

[77] Meyer A.J., Dreyer A., Ugalde J.M., Feitosa-Araujo E., Dietz K.-J., Schwarzländer M. (2021). Shifting paradigms and novel players in Cys-based redox regulation and ROS signaling in plants—And where to go next. Biol. Chem..

[78] Dixon D.P., Skipsey M., Grundy N.M., Edwards R. (2005). Stress-induced protein *s*-glutathionylation in Arabidopsis. Plant Physiol..

[79] Durek P., Schmidt R., Heazlewood J.L., Jones A., MacLean D., Nagel A., Kersten B., Schulze W.X. (2009). PhosPhAt: The *Arabidopsis thaliana* phosphorylation site database. An update. Nucleic Acids Res..

[80] Tossounian M.-A., Van Molle I., Wahni K., Jacques S., Gevaert K., Van Breusegem F., Vertommen D., Young D., Rosado L.A., Messens J. (2018). Disulfide bond formation protects *Arabidopsis thaliana* glutathione transferase tau 23 from oxidative damage. Biochim. Et Biophys. Acta (BBA)—Gen. Subj..

[81] Tossounian M.A., Wahni K., Van Molle I., Vertommen D., Astolfi Rosado L., Messens J. (2018). Redox-regulated methionine oxidation of *Arabidopsis thaliana* glutathione transferase Phi9 induces H-site flexibility. Protein Sci..

[82] Mittler R., Zandalinas S.I., Fichman Y., Van Breusegem F. (2022). Reactive oxygen species signalling in plant stress responses. Nat. Rev. Mol. Cell Biol..

[83] Huang S., Gao Y., Liu J., Peng X., Niu X., Fei Z., Cao S., Liu Y. (2012). Genome-wide analysis of WRKY transcription factors in *Solanum lycopersicum*. Mol. Genet. Genom..

[84] Gao Y., Liu J., Yang F., Zhang G., Wang D., Zhang L., Ou Y., Yao Y. (2019). The WRKY transcription factor WRKY8 promotes resistance to pathogen infection and mediates drought and salt stress tolerance in *Solanum lycopersicum*. Physiol. Plant..

[85] Sun X.-c., Gao Y.-f., Li H.-r., Yang S.-z., Liu Y.-s. (2015). Over-expression of *SlWRKY39* leads to enhanced resistance to multiple stress factors in tomato. J. Plant Biol..

[86] Karkute S.G., Gujjar R.S., Rai A., Akhtar M., Singh M., Singh B. (2018). Genome wide expression analysis of WRKY genes in tomato (*Solanum lycopersicum*) under drought stress. Plant Gene.

[87] Hichri I., Muhovski Y., Clippe A., Žižková E., Dobrev P.I., Motyka V., Lutts S. (2015). SlDREB2, a tomato dehydration-responsive element-binding 2 transcription factor, mediates salt stress tolerance in tomato and Arabidopsis. Plant Cell Environ..

[88] Thirumalaikumar V.P., Devkar V., Mehterov N., Ali S., Ozgur R., Turkan I., Mueller-Roeber B., Balazadeh S. (2017). NAC transcription factor Jungbrunnen1 enhances drought tolerance in tomato. Plant Biotechnol. J..

[89] Jiang L., Wang Y., Zhang S., He R., Li W., Han J., Cheng X. (2016). Tomato *SlDREB1* gene conferred the transcriptional activation of drought-induced gene and an enhanced tolerance of the transgenic arabidopsis to drought stress. Plant Growth Regul..

[90] Hichri I., Muhovski Y., Žižková E., Dobrev P.I., Gharbi E., Franco-Zorrilla J.M., Lopez-Vidriero I., Solano R., Clippe A., Errachid A. (2017). The *Solanum lycopersicum* WRKY3 transcription factor SlWRKY3 is involved in salt stress tolerance in tomato. Front. Plant Sci..

[91] Chinnapandi B., Bucki P., Fitoussi N., Kolomiets M., Borrego E., Braun Miyara S. (2019). Tomato SlWRKY3 acts as a positive regulator for resistance against the root-knot nematode *Meloidogyne javanica* by activating lipids and hormone-mediated defense-signaling pathways. Plant Signal. Behav..

[92] Du M., Zhao J., Tzeng D.T.W., Liu Y., Deng L., Yang T., Zhai Q., Wu F., Huang Z., Zhou M. (2017). MYC2 orchestrates a hierarchical transcriptional cascade that regulates jasmonate-mediated plant immunity in tomato. Plant Cell.

[93] Bhattarai K.K., Atamian H.S., Kaloshian I., Eulgem T. (2010). WRKY72-type transcription factors contribute to basal immunity in tomato and Arabidopsis as well as gene-for-gene resistance mediated by the tomato R gene Mi-1. Plant J..

