# Supplementary material for: Glutathione Transferases Are Involved in the Genotype-Specific Salt-Stress Response of Tomato Plants
Source: Antioxidants (Basel). 2023 Aug 28;12(9):1682. doi: 10.3390/antiox12091682 (PMC10525892; doi:10.3390/antiox12091682)
Supplement: Supplementary file 1 [file antioxidants-12-01682-s001.zip › Spplementary Figures.pdf]

## Supplementary Figures

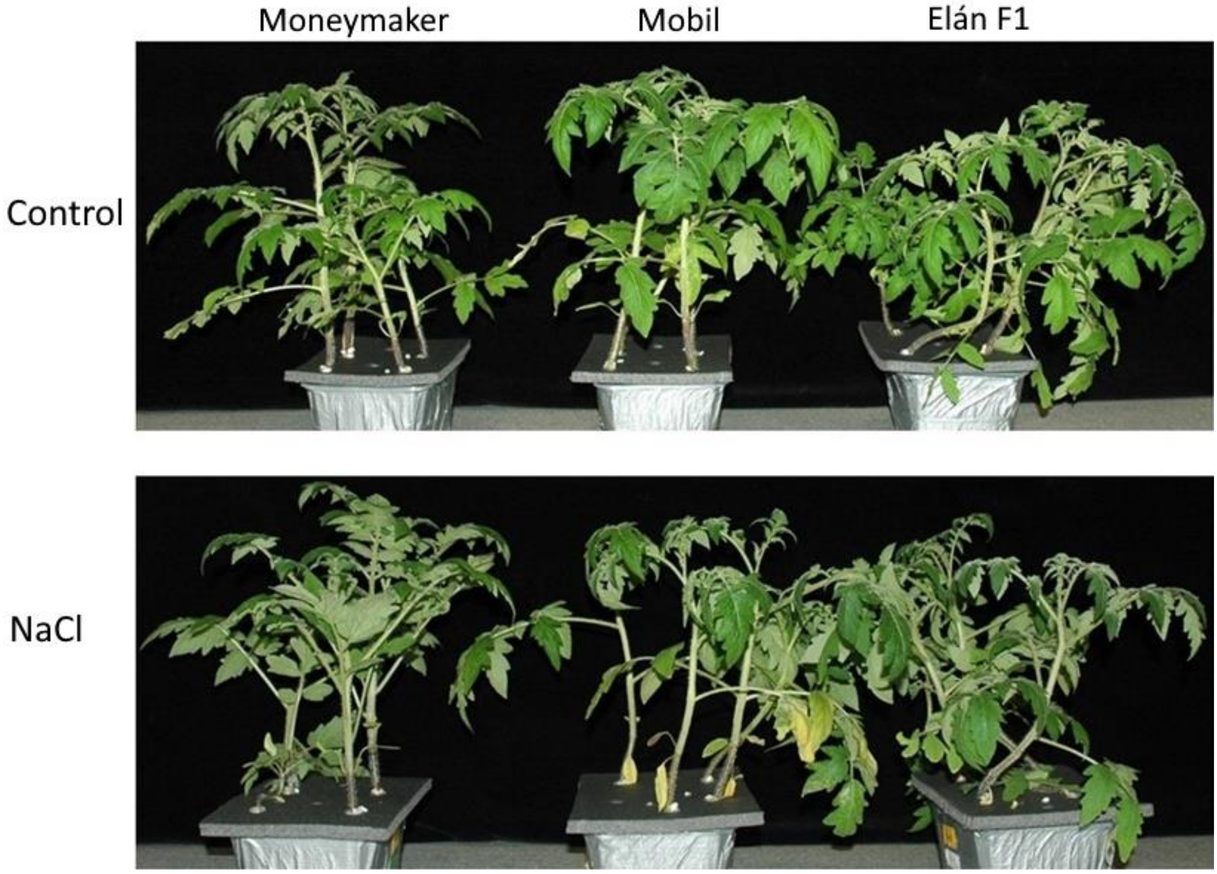


**Figure S1.** Image of five-week-old tomato plants (*Solanum lycopersicum* L., Moneymaker, Mobil and Elán F1 cultivars) grown in hydroponic culture under control conditions (Control) and after one week of 100 mM NaCl treatment (NaCl).

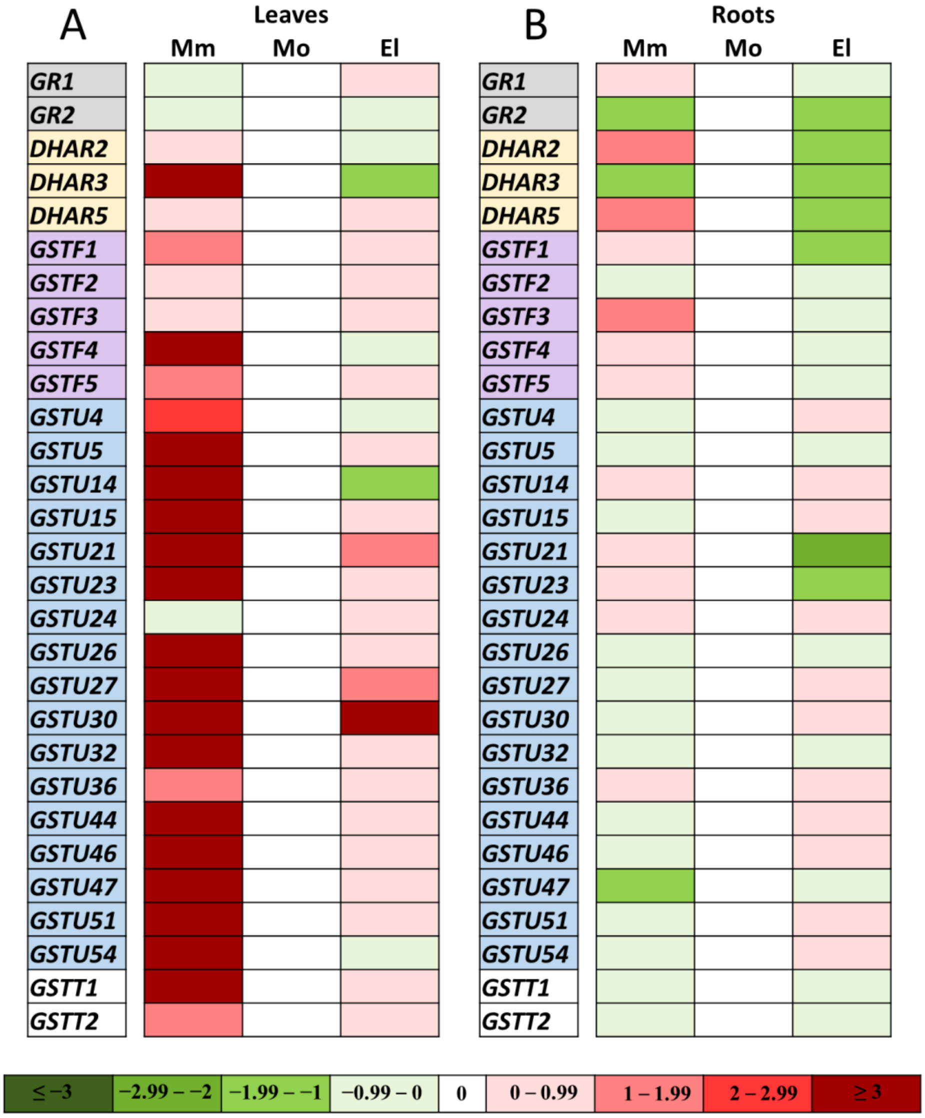


**Figure S2.** Heat map of the expression levels of two *Solanum lycopersicum* glutathione reductases (*GR1*, *GR2*) and 27 selected genes belonging to four GST classes (*DHAR*, *GSTF*, *GSTU* and *theta GST*) determined in the leaves (A) and roots (B) of five-week-old control Moneymaker (Mm), Mobil (Mo) and Elán F1 (El) tomato cultivars. Relative transcript amounts of the genes in leaves and roots were determined by HT-qPCR. The expression of genes was normalized fist by the average of *actin2* and *elongation factor 1α* genes, and second to the average transcript amount of each gene in untreated Mobil cultivar under control condition. Green colours show repression, while red colours show activation, as it is indicated in the colour scale bar. Log$_2$ transformations of $2^{-\Delta\Delta Ct}$ data were presented in the heat map. The presented data are from two biological replicates.

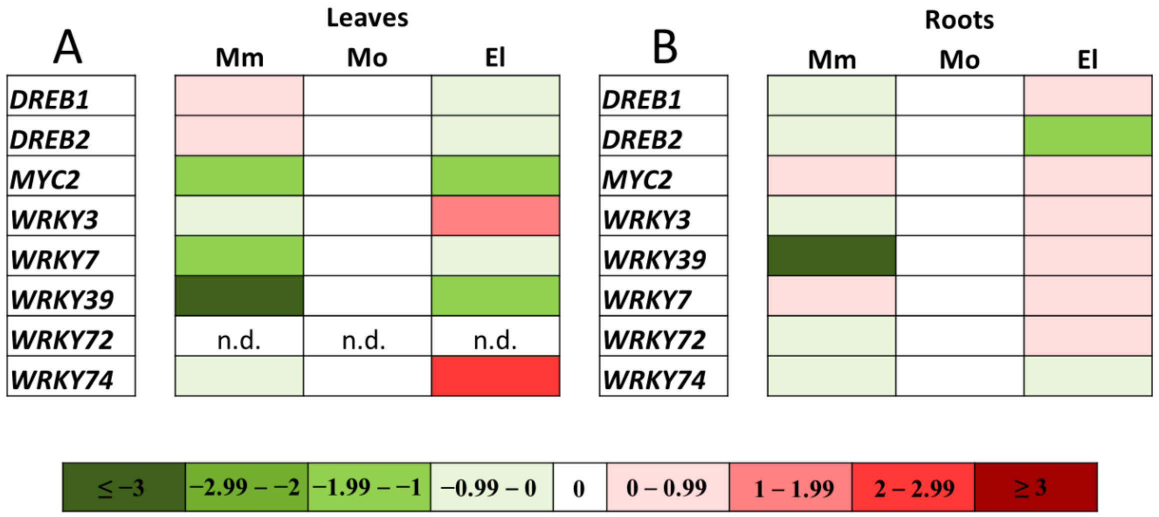


**Figure S3.** Heat map of eight *Solanum lycopersicum* transcription factors gene expression. Relative transcript amounts of genes were determined in five-week-old control tomato (Moneymaker - Mm, Mobil - Mo and Elán F1 - El) leaves (A) and roots (B) by qPCR. The expression of genes was normalized first by the *actin2* gene, and second to the average transcript amount of each gene in untreated Mobil cultivar. $Log_2$ transformations of $2^{-\Delta\Delta Ct}$ data were presented as a heat map. Green colour represents repression, while red colour represents activation, as it is indicated on the upper colour scale bar. The presented data are from two biological replicates. n.d. – not detected
